# Asymmetric Load Transmission Induces Facet Joint Subchondral Sclerosis and Hypertrophy in Patients with Idiopathic Adolescent Scoliosis: Evaluation Using Finite Element Model and Surgical Specimen

**DOI:** 10.1002/jbm4.10812

**Published:** 2023-08-29

**Authors:** Yasuhito Yahara, Shoji Seki, Hiroto Makino, Hayato Futakawa, Katsuhiko Kamei, Yoshiharu Kawaguchi

**Affiliations:** ^1^ WPI‐Immunology Frontier Research Center Osaka University Suita Japan; ^2^ Department of Orthopaedic Surgery, Faculty of Medicine University of Toyama Toyama Japan

**Keywords:** ADOLESCENT IDIOPATHIC SCOLIOSIS, FACET JOINT, FINITE ELEMENT ANALYSIS, SUBCHONDRAL BONE SCLEROSIS, THORACIC SPINE

## Abstract

Adolescent idiopathic scoliosis (AIS) with thoracic curvature primarily progresses from the thoracolumbar region, causing abnormal twisting and rotation of the spinal column. This results in unbalanced, asymmetric loads on each vertebrae and increased demands on the thoracic facet joints to withstand rotational stress from adjacent vertebrae. However, no studies have focused on the stress distribution on the facet joints of the thoracic spine in patients with AIS. This study aimed to investigate the mechanical loading and its distribution on the thoracic facet joints of AIS patients using finite element (FE) analysis and surgical specimens. FE models of the thoracic spine were created from a total of 13 female AIS patients (Lenke type 1, *n* = 4; Lenke type 2, *n* = 4; Lenke type 3, *n* = 5). A load of 200 N on the T3 vertebrae and 30 N each on the bilateral superior articular processes were applied vertically to quantify the contact force on the facet joints from T3 to T11. In addition, morphological and histological analyses were performed on the inferior articular processes obtained during surgery. FE analysis demonstrated that contact forces of the facet joint progressively increased from the mid to lower thoracic spine of the concave side, reaching a maximum around the apex. More than 91% of the load was transmitted by the facet joints at the concave side, resulting in facet joint subchondral sclerosis and hypertrophy. The apical facet joint in AIS helps counteract rotational stress between vertebrae and transfers most stress through the concave side. In conclusion, this study found that asymmetric load transfer in the facet joints leads to subchondral sclerosis and hypertrophy. These findings can enhance our understanding of the stress loading on facet joints and the resulting biological changes and help clarify the mechanisms involved in scoliosis progression. © 2023 The Authors. *JBMR Plus* published by Wiley Periodicals LLC on behalf of American Society for Bone and Mineral Research.

## Graphical abstract

Finite element models reveal the asymmetric stress loading and distribution on the thoracic facet joints of adolescent idiopathic scoliosis. The asymmetric load transmission could be responsible for hypertrophy and subchondral sclerosis of the facet joint of adolescent idiopathic scoliosis.

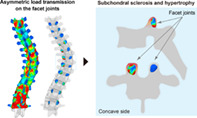

## Introduction

The spinal column supports our bodies with a three‐dimensional (3D) structure and shape. The primary functions of the spine are to protect the spinal cord and nervous system and to transfer mechanical load from the trunk to the pelvis. The physiological load transfer across the spine plays a role in maintaining musculoskeletal health and preventing disease. Conversely, deterioration of spinal alignment can lead to muscle stiffness and chronic back pain caused by the development of osteoarthritis and degeneration of the spine. The vertebral body and intervertebral disc symmetrically transmit 70% to 80% of the load, and the facet joint compensates for the remaining 20% to 30% of the distribution in normal individuals.^(^ ^1^ ^)^ However, spinal malalignment, which occurs in adolescent idiopathic scoliosis (AIS), can alter this physiologic load transfer.

AIS is one of the significant spinal deformities that predominantly occur in girls between 10 and 13 years, characterized by sideward curvature and rotation of the vertebrae.^(^ ^2^ ^)^ The risk of curve progression occurs most frequently during the prepubertal period of peak height velocity.^(^ ^3^, ^4^ ^)^ This rapid curve progression and deformation of the spine during the growing period is known to be triggered by asymmetric mechanical stresses on the spine that causes wedging of the intervertebral discs and vertebrae.^(^ ^5^, ^6^, ^7^, ^8^ ^)^ This phenomenon might be explained by the Hueter–Volkmann Law,^(^ ^9^ ^)^ which states that bone growth during skeletal immaturity is delayed by mechanical compression of the growth plate and accelerated by the tension of the growth plate.^(^ ^10^, ^11^, ^12^ ^)^ Moreover, previous reports suggested that scoliosis curvature was based on a combination of disc and vertebral wedging.^(^ ^6^, ^13^ ^)^

Finite element (FE) analysis allows us to analyze intricate boundary conditions and loading. In recent advancements in this field, several biomechanical studies employing FE analysis have provided valuable insights into the muscle moment arms associated with dynamic joint motion and mechanical loading in various joints, including the shoulder,^(^ ^14^ ^)^ lower extremity,^(^ ^15^ ^)^ and foot.^(^ ^16^ ^)^ However, the spinal column comprises multiple contiguous vertebrae flanked by intervertebral discs and facet joints. In addition, complex 3D anatomy, which includes various components such as muscles and ligaments, poses a significant challenge to applying FE modeling to scoliosis. Previous studies used FE analysis to examine the detailed stress distribution on the scoliotic spine. Nonetheless, these studies either examined the mechanical loading on the lumbar facet joint in degenerative scoliosis^(^ ^17^ ^)^ or focused mainly on stress on the vertebral bodies and discs.^(^ ^18^, ^19^, ^20^ ^)^ Since AIS progression typically initiates in the thoracic spine, investigating the stress distribution in this region can lead to a better understanding of AIS etiology. As scoliosis advances, spinal curvature and rotational stresses between vertebrae and facet joints are expected to increase. Therefore, the thoracic spine of patients with AIS should be modeled using the FE method, and stress analysis of the facet joints should be performed.

Facet joints are symmetrical synovial joints covered with fibrous capsules that protect motion from anterior shear forces, excessive rotation, and flexion. However, the facet joint undergoes degenerative changes due to excessive mechanical loading, age‐related wear, and trauma.^(^ ^21^ ^)^ This condition is termed facet joint osteoarthritis or facet arthropathy.^(^ ^22^ ^)^ Bisson et al. demonstrated that articular cartilage of AIS showed characteristics of osteoarthritis, including proteoglycan loss, overexpression of inflammatory mediators, and increased synthesis of matrix‐degrading proteases.^(^ ^1^ ^)^ Furthermore, thoracic kyphosis is compensatory decreased in patients with AIS,^(^ ^23^ ^)^ resulting in increased mechanical loading on the facet joints. Although the asymmetrical mechanical loading on the facet joint in AIS is a potential trigger for scoliosis‐related low back pain and osteoarthritis, poor biomechanical evidence exists regarding stress distribution on the facet joints in patients with AIS.

This study aimed to investigate the mechanical load distribution of the thoracic facet joints of AIS. To achieve this objective, FE models were created from the thoracic spine using computed tomography (CT) images of patients with AIS of different curve types. We also attempted to confirm the pathological changes in the surgical specimen of the facet joints. The stress distribution revealed by the FE model reflected clinical and histological findings based on facet degeneration, such as thickening of the facet joints and subchondral osteosclerosis. These data provide insight into the details of stress loading of the thoracic facet joints of AIS patients.

## Materials and Methods

### Participants

This study was approved by the ethics committee of Toyama University Hospital and performed in accordance with the ethical guidelines of the committee. Patients and their legal guardians provided written informed consent for participation in the analysis. A total of 13 female AIS patients with thoracic curvature (Lenke type 1, *n* = 4; Lenke type 2, *n* = 4; Lenke type 3, *n* = 5) who underwent scoliosis correction and fixation surgery at our institution were included in the study. The mean age at surgery was 13.2 ± 1.4 years, and the mean thoracic Cobb angle was 55.0 ± 9.4^o^. No statistical differences were observed in age, Cobb angle, thoracic kyphosis, height, body weight, level of apical vertebrae, and Risser grade among Lenke type 1, 2, and 3 patients (Table 1).

**Table 1:** Demographics

	Overall (*n* = 13)	Lenke type 1 (*n* = 4)	Lenke type 2 (*n* = 4)	Lenke type 3 (*n* = 5)	*p* value
Age	13.2 ± 1.4	12.8 ± 1.0	13.8 ± 1.7	13.2 ± 1.8	0.87
Height (cm)	155.3 ± 4.3	154.6 ± 3.0	155.5 ± 5.8	155.3 ± 5.5	0.93
Body weight (kg)	46.0 ± 6.0	41.5 ± 2.8	45.4 ± 5.1	49.9 ± 7.3	0.089
Cobb angle (degree)	55.0 ± 9.4	50.5 ± 7.6	56.8 ± 12.4	57.4 ± 8.8	0.67
Thoracic kyphosis (degree)	21.4 ± 9.7	25.2 ± 10	23.0 ± 4.0	17.0 ± 12.3	0.25
Apex level					
T8	4	1	0	3	0.25
T8/9	5	1	3	1	
T9	4	2	1	1	
Risser sign (grade)					
0	0	0	0	0	0.55
1	0	0	0	0	
2	3	1	1	1	
3	6	3	1	2	
4	4	0	2	2	
5	0	0	0	0	

To reduce variation in bone strength, this study included patients with bone maturity lower than Risser sign 4. Additionally, this study included patients with the apical vertebrae of the main thoracic curve located between T8 and T9 to minimize the influence of the curve pattern variation on the results. We did not formally verify the sample size required for statistical analysis. Instead, the number of samples that could be collected at our facility was used for the analysis.

### Model construction and material properties

The FE model was generated using FE analysis software to quantify the distribution of mechanical loading on the spinal column (MECHANICAL FINDER, Research Center of Computational Mechanics, Tokyo, Japan). Preoperative CT scans with a 0.75‐mm slice distance of the entire spine obtained from AIS patients were applied to MECHANICAL FINDER version 10.0. Based on the CT values, we extracted the cortical bone from T3 to L2 vertebrae, including anatomical landmarks such as the vertebral body, endplates, transverse processes, spinous processes, and upper and lower facet joint articular surfaces. To accurately depict the intervertebral discs and facet joints, we created their outlines by interpolating the profiles of the upper and lower border endplates and the subchondral bone. The spinal component, including the vertebrae, intervertebral disc, and facet joints, were meshed using linear tetrahedral elements with a 2.5‐mm global size and 0.3125‐mm minimum size. The Young's modulus of the vertebrae was obtained by the equation proposed by Keyak et al.,^(^ ^24^ ^)^ and Poisson's ratio was set at 0.4. Young's moduli and Poisson's ratios for the intervertebral discs (4.2 MPa and 0.45, respectively)^(^ ^25^ ^)^ and articular cartilage of facet joints (20 MPa and 0.3, respectively)^(^ ^26^, ^27^ ^)^ were used as previously reported (Table 2).

**Table 2:** Material properties

	Young's modulus	Poisson's ratio
Thoracic and lumbar vertebrae	Formula proposed by Keyak^24^	0.4
Intervertebral discs	4.2 Mpa	0.45
Facet joints	20 Mpa	0.3

### Loading and boundary condition

**Fig. 1:**
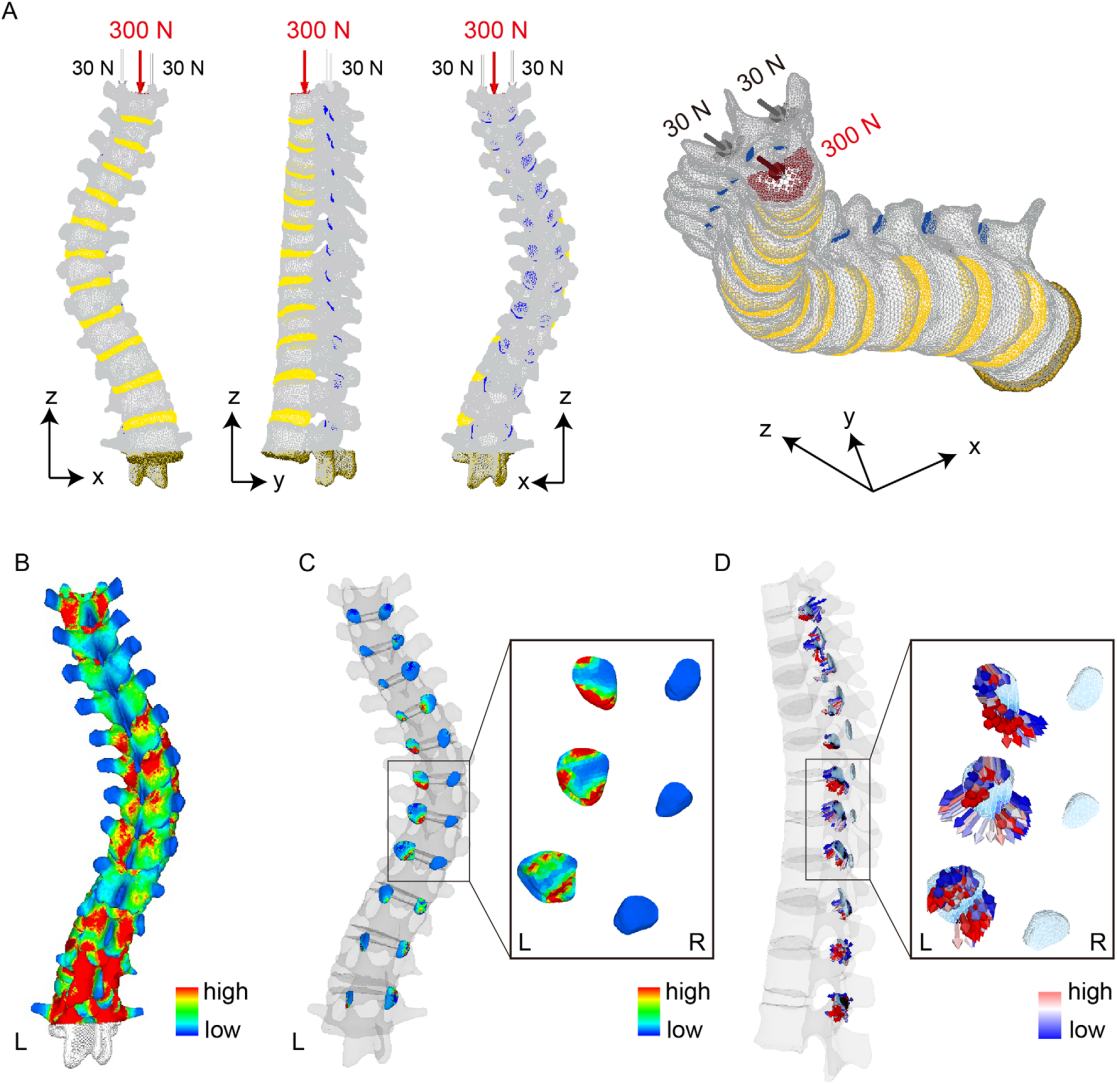
Schematic representation of mechanical loading and boundary conditions. A load of 200 N on the T3 vertebrae, with 30 N on each bilateral superior articular processes, was applied in the vertical direction. During loading, the L2 vertebrae was fully fixed, while the T3 vertebrae was restrained along the X and Y axes and kept free only along the Z axis (*A*). Histogram showing distribution intensity of contact force at each facet joint (*B*, *C*). Vector diagram indicating stress direction (*D*).

Previous reports showed that the upper body's weight accounts for 54% to 66% of the total body weight.^(^ ^28^, ^29^ ^)^ In this study, a load of 260 N, corresponding to 57% of the average patient's body weight (46.0 kg), was applied vertically to the T3 vertebrae and bilateral superior articular processes. Vertebral bodies and intervertebral discs evenly distribute around 70% to 80% of the load, while the remaining 20% to 30% is transmitted by the facet joints. Consequently, a load of 200 N was applied to the T3 vertebral body and 30 N each to the bilateral superior articular processes. During loading, the L2 vertebrae was fully fixed, while the T3 vertebrae was restrained in the X and Y axes and kept free only in the Z axis (Fig. 1*A*). In previous FE analyses using the lumbar spine, various loading conditions, such as flexion, extension, and rotation, were applied at the cranial endplate to evaluate the stress distribution of the spinal column.^(^ ^30^, ^31^ ^)^ However, in this study, we focused solely on the stress distribution of the facet joints in response to vertical loading to the T3 vertebrae. The contact conditions were defined between the inferior articular processes and the articular cartilages with a friction coefficient of 0.0. The articular cartilage was assumed to be attached to the upper articular process, and contact conditions were established between the articular cartilage and the lower articular process. The total contact force (N) at each facet joint was quantified (Fig. 1*B*–*D*).

### Thickness measurement of the inferior articular process

Based on the preoperative CT scan results, the thickness of the inferior articular process was measured from T1 to T11 using SYNAPSE VINCENT medical imaging system (Fujifilm) to evaluate the effect of asymmetric loading on the facet joint. Delta thickness was defined by subtracting the thickness of the left inferior articular process from the right side. In some experiments, Hounsfield unit (HU) values of the inferior articular process were measured using the round region‐of‐interest tool of SYNAPSE VINCENT and compared between the concave and convex sides.

### Histological analysis

The inferior articular processes with the highest delta thickness values were removed bilaterally during corrective spinal fusion surgery. Dissected inferior articular processes were fixed in 4% paraformaldehyde overnight and decalcified in 14% EDTA for 4 weeks. Histological analysis was performed with hematoxylin and eosin staining.

### Statistical analysis

**Fig. 2:**
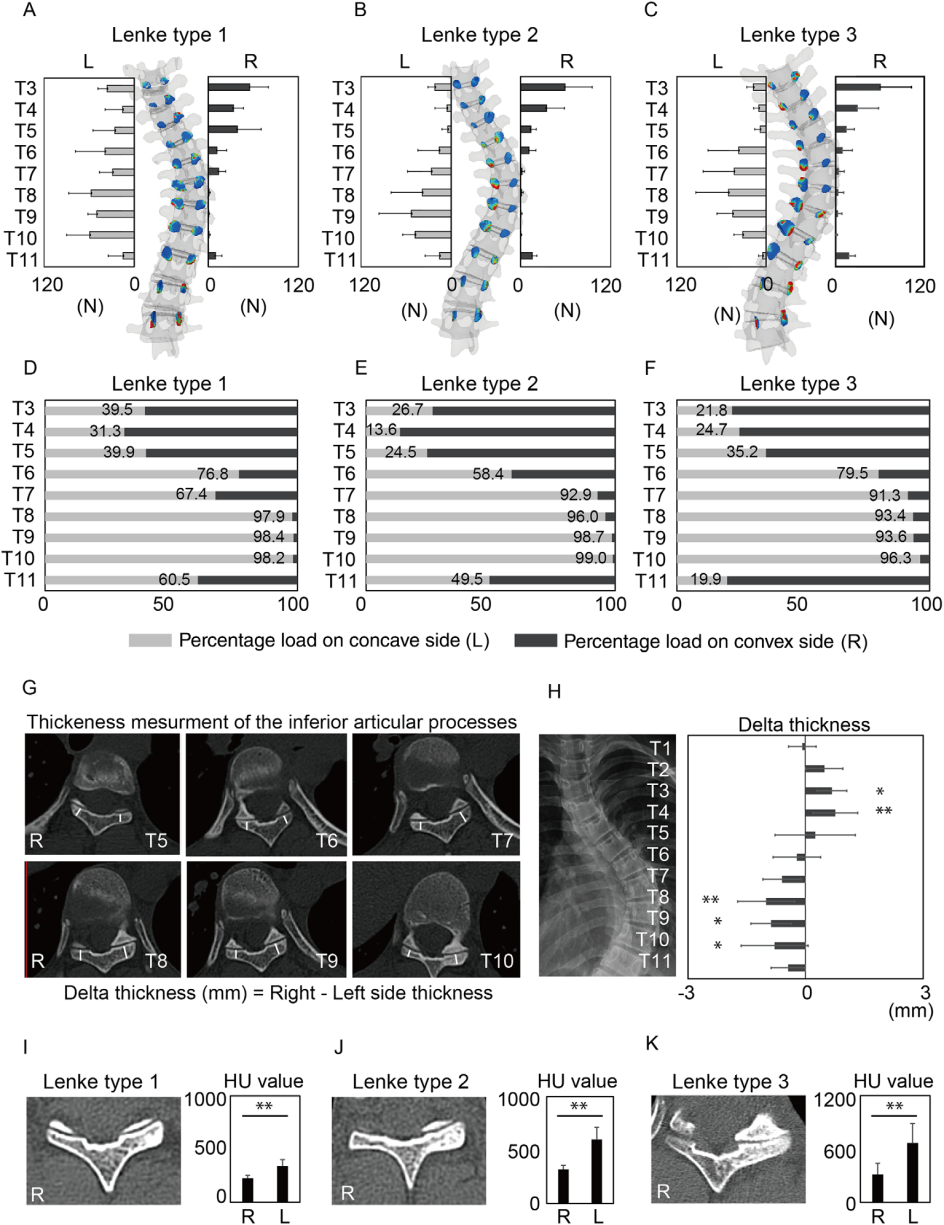
Asymmetric load transfer and subchondral bone sclerosis. Contact forces at each facet joint in Lenke types 1 (*A*), 2 (*B*), and 3 (*C*). The gray bars indicate the percentage of load transmitted to the concave side, and the black bars indicate the percentage of load transmitted to the convex side of the facet joint in Lenke type 1 (*D*), Lenke type 2 (*E*), and Lenke type 3 (*F*). Representative CT images of inferior articular process at each facet joint (*G*). Delta thickness, defined by subtracting the thickness of the left inferior articular process from the right side, was measured from T1 to T11 (*H*). Statistical analysis was performed using ANOVA followed by Dunnett's multiple comparisons test. *p < 0.05; **p < 0.01 compared with T1. Data are presented as the mean ± SD. Representative CT images of subchondral sclerosis and quantitative Hounsfield unit (HU) values of the inferior articular process in Lenke types 1 (*I*), 2 (*J*), and 3 (*K*). Statistical analysis was performed using unpaired two‐tailed *t* tests. **p < 0.01. Data are presented as mean ± SD.

Comparison of age, height, weight, Cobb angle, and thoracic kyphosis angle among Lenke types 1, 2, and 3 was carried out using ANOVA followed by Tukey's multiple comparison test. Comparisons of apex level and Risser grade between Lenke types 1, 2, and 3 were assessed using the chi‐squared test (Table 1). In addition, delta thickness of the inferior articular process (in millimeters) was assessed using ANOVA followed by Dunnett's multiple comparison test (Fig. 2*H*). The contact forces (Newton) occurring at each facet joint were compared using a *t* test (two‐sided, Table 3). The data were presented as means and SDs. Statistical analyses were performed using GraphPad Prism (GraphPad Software, San Diego, CA, USA). Statistical significance was set at *p* < 0.05.

**Table 3:** Contact forces (Newton) from T3 to T11 facet joints in Lenke types 1, 2, and 3

	Lenke type 1			Lenke type 2			Lenke type 3		
	L (N)	R (N)	*p* value	L (N)	R (N)	*p* value	L (N)	R (N)	*p* value
T3	36.1 ± 12.1	55.3 ± 24.7	0.213	21.6 ± 9.2	59.3 ± 36.1	0.082	17.0 ± 8.9	61.0 ± 41.5	0.049
T4	15.4 ± 23.3	33.6 ± 13.3	0.222	5.4 ± 16.0	34.6 ± 23.7	0.054	9.8 ± 6.9	29.7 ± 28.3	0.165
T5	25.8 ± 29.2	38.7 ± 31.6	0.569	4.5 ± 7.7	13.8 ± 6.8	0.099	7.9 ± 7.3	14.6 ± 10.6	0.282
T6	38.9 ± 39.4	11.7 ± 12.7	0.237	15.9 ± 28.3	11.3 ± 9.0	0.722	36.9 ± 41.4	9.3 ± 13.2	0.193
T7	28.6 ± 17.8	13.8 ± 9.4	0.191	26.3 ± 31.7	1.9 ± 3.3	0.034	43.1 ± 41.7	4.0 ± 7.5	0.073
T8	57.4 ± 29.8	1.1 ± 1.6	0.009	38.3 ± 41.6	1.5 ± 2.1	0.010	50.6 ± 44.0	3.5 ± 7.2	0.046
T9	49.8 ± 11.9	0.7 ± 0.6	<0.001	53.0 ± 43.0	0.6 ± 1.3	0.002	44.9 ± 21.3	3.0 ± 5.4	0.003
T10	59.3 ± 30.7	1.0 ± 1.4	0.009	47.8 ± 16.5	0.4 ± 0.9	0.001	31.3 ± 12.0	1.2 ± 1.1	<0.001
T11	14.8 ± 19.6	9.6 ± 8.4	0.646	15.4 ± 19.2	15.7 ± 6.1	0.981	4.5 ± 5.4	18.2 ± 8.3	0.015

*Note*: Statistical analysis was performed using unpaired two‐tailed *t* tests. Data are mean ± SD. Statistical significance was set at *p* < 0.05.

## Results

FE analysis showed that the contact forces progressively increased from the mid‐thoracic to the lower‐thoracic spine, reaching a maximum in the concave T8 to T10 facet joint in all Lenke types (Fig. 2*A*–*C*). In the Lenke type 1 curve, more than 95% of the mechanical load was transmitted through the facet joints at the concave side from T8 to T10 (Fig. 2*D*). This trend was consistent for Lenke types 2 and 3, with more than 91% of the load distributed over the facet joints at the concave side from T7 to T10 (Fig. 2*E*,*F*). In contrast, the facet joints of the upper thoracic spine from T3 to T5 transmitted the load mainly through the convex side. Since the apical vertebrae of all patients were located at T8 to T9, the load distribution of the facet joints was shown to be significantly shifted to the concave side around the apical vertebrae (Table 3).

**Fig. 3:**
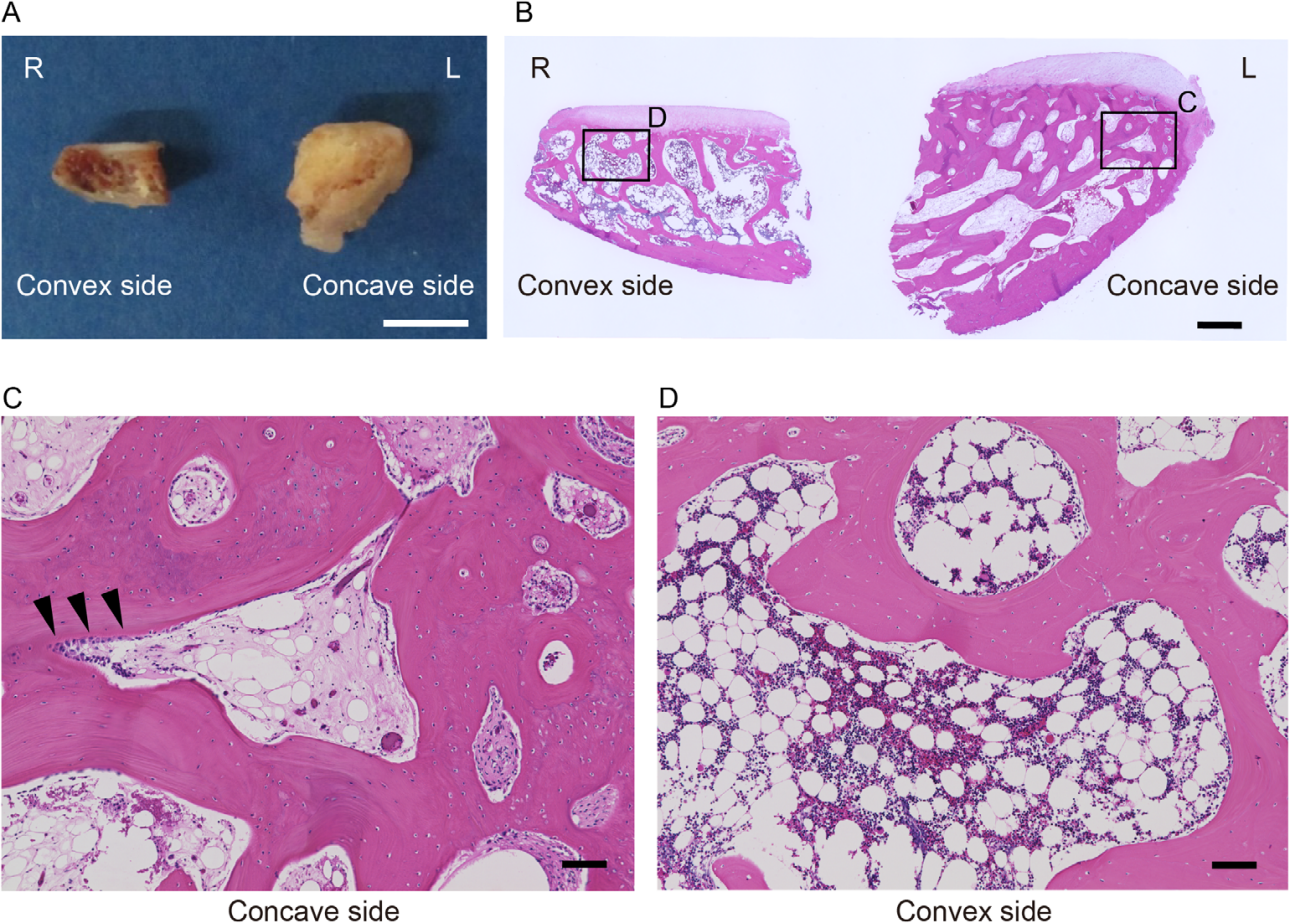
Morphological and histological findings of subchondral bone sclerosis and hypertrophy in inferior articular process. Representative gross findings of subchondral osteosclerosis and hypertrophy in inferior articular process of AIS patients (*A*). Hematoxylin and eosin staining showed increased trabecular bone thickness in the subchondral bone on the concave side (*B*, *C*) and decreased trabecular bone thickness on the convex side (*B*, *D*). Osteoblasts (black triangles) and lining cells are observed in the subchondral bone on the concave side, reflecting active bone remodeling. Scale bar: (*A*) 5 mm; (*B*) 1 mm; (*D*) 100 μm.

To evaluate the effect of asymmetric load distribution on the facet joint, delta facet thickness (defined by subtracting the thickness of the left inferior articular process from the right side) was measured from T1 to T11 using a CT image (Fig. 2*G*). The facet thickness increased continuously on the convex side from T1 to T4 and began to decrease at the T5 level. Similarly, the thickness on the concave side increased from T7 onward, reaching a peak at T8 (Fig. 2*H*). This result was comparable to the load distribution pattern observed in the FE analysis. In addition, HU values of the inferior articular process were also measured to evaluate stress‐induced bony sclerosis. HU values of the subchondral bone were significantly elevated on the concave side, representing subchondral sclerosis of the inferior articular process due to excessive mechanical loading in all curve types (Fig. 2*I*–*K*). Morphological and histological analysis also revealed significant osteosclerotic findings in the inferior articular process on the concave side (Fig. 3*A*). Furthermore, the facet joints on the concave side were thicker than those on the convex side, with increased trabecular bone thickness (Fig. 3*B*,*C*). In contrast, the convex side had decreased trabecular bone thickness and increased bone marrow adipose tissue (Fig. 3*B*,*D*). In response to asymmetric mechanical loading, osteoblasts and lining cells were observed in the subchondral bone on the concave side, presumably reflecting activated bone remodeling (Fig. 3*C*). These findings demonstrate that the apical facet joint of AIS helps counteract rotational stress between vertebrae and transfers the majority of stress through the concave side, leading to facet joint subchondral sclerosis and hypertrophy.

## Discussion

Rapid progression of spinal curvature during the adolescent growth spurt leads to complex 3D deformity of the spine and trunk in AIS. In this study, we quantified the asymmetric stress loading and distribution on the facet joints of AIS patients using FE analysis. Most of the mechanical load through the posterior column of the spine was transmitted by the facet joints at the concave side, resulting in facet joint subchondral sclerosis and hypertrophy.

Most existing studies regarding the morphological features of scoliosis focus on wedging of the intervertebral discs and vertebrae, pedicle, and facet joint based on X‐ray, CT scan, and magnetic resonance imaging findings.^(^ ^6^, ^12^, ^13^, ^32^, ^33^, ^34^, ^35^ ^)^ Wedging of the vertebral bodies is more pronounced around the apical vertebrae (from T7 to T10),^(^ ^12^ ^)^ with lower vertebral body height on the concave side compared to the convex side.^(^ ^3^, ^6^, ^7^, ^8^ ^)^ In addition, the pedicle cross‐sectional area, width, and height on the concave side are reduced compared to the convex side around the apex.^(^ ^6^, ^12^ ^)^ Moreover, especially in T8 and T12 of the lower thoracic spine, the superior facet joint areas are approximately 2.3 times larger on the concave side than on the convex side.^(^ ^12^ ^)^ These previous findings were possibly due to the fact that the concave side of scoliosis is exposed to a more significant load. However, mechanobiology studies that adequately test this hypothesis are limited. In this study, FE analysis provides evidence that contact forces of the facet joint gradually increase from the mid to lower thoracic region, reaching a maximum at the concave T8 to T10 facet joint.

In addition to hypertrophy of the facet joint, subchondral bone sclerosis was observed in the inferior articular process on the concave side. According to Wolff's law,^(^ ^36^ ^)^ bone is mechanosensitive and thus can change its volume, structure, and mechanical properties in response to mechanical loading. Since hypertrophy and subchondral sclerosis correlated with the contact force of the facet joints, it was considered to be the result of increased asymmetric loading. When mechanical loading exceeds the physiological range, it can lead to joint surface damage and osteoarthritis development. The facet joints of AIS patients are more degenerated on the concave side, suggesting that the younger facet joint of AIS may have similar alterations to patients with facet joint osteoarthritis.^(^ ^1^ ^)^ Facet joint osteoarthritis is a clinical and pathological manifestation of the synovial facet joint. Narrowing of the joint surfaces, subarticular bone erosions, subchondral sclerosis, osteophyte formation, and hypertrophy of the articular processes are typical radiographic features of facet joint osteoarthritis.^(^ ^37^ ^)^ Although facet joint osteoarthritis is a possible cause of chronic back pain,^(^ ^38^, ^39^, ^40^ ^)^ little is known regarding its involvement in the pathophysiology of AIS. Thus, further research is required to determine how facet joint alterations due to load imbalance impact the development and progression of AIS.

The “vicious cycle” model is a widely accepted explanation for the mechanism of scoliosis progression.^(^ ^10^ ^)^ Asymmetric load transmission in scoliosis can cause vertebral and intervertebral disc wedging and vertebral overgrowth, resulting in further progression of scoliosis. An additional hallmark of progressive scoliosis is hypokyphosis of the thoracic spine.^(^ ^23^ ^)^ Hypokyphosis can increase load transmission in the posterior part of the spine and enhance anterior traction forces on the vertebral bodies, facilitating vertebral body overgrowth.^(^ ^41^ ^)^ In addition, increased asymmetric loading on the facet joints can contribute to reduced trunk flexion capacity, which may further contribute to the progression of thoracic hypokyphosis. Thus, wedging of the vertebral bodies and discs, overgrowth of the vertebral bodies, hypokyphosis, and load imbalance to the facet joints can all contribute to a “vicious cycle” and accelerate scoliosis.

In this study, the load distribution on the facet joints was analyzed using the FE method for AIS patients with different curve patterns of Lenke types 1, 2, and 3. However, the distribution pattern of the mechanical load was quite similar among all curve types. This was because the FE model was limited to the thoracolumbar region from T3 to L2, and the analysis focused only on the thoracic spine from T3 to T11. Since scoliosis is a deformity of the entire spine from the head to the pelvis, it seemed necessary to consider the whole spine to evaluate the mechanical load distribution across the different curve types.

Replicating the loading conditions of the scoliotic spine is extremely challenging for several reasons. First, the spine is mainly stabilized by the complex interplay of muscles, ligaments, and ribs. Second, scoliosis involves 3D deformities of the spine, including kyphosis, lordosis, rotation, and torsion, which make the distribution of mechanical pressure more complex than in healthy individuals. Patwardhan et al. proposed follower loading, which applies compressive loads close to the tangent of the lumbar kyphosis direction using human cadaver spine specimens. They discovered that follower loading could increase the load‐bearing capacity of the lumbar spine compared to vertical loading.^(^ ^42^ ^)^ In this study, we only evaluated the vertical loading on the thoracic spine. Future research should aim to validate the direction of loading and develop a model that includes ligaments, muscles, and ribs. Additionally, the validity of the model should be evaluated using spinal specimens from cadavers of patients with scoliosis.

The combination of FE methods and musculoskeletal models offers numerous possibilities. Recent studies have highlighted the capability of FE models to analyze the mechanical effects on the body under various loading conditions and movements.^(^ ^16^, ^43^ ^)^ Moreover, by incorporating patient‐specific anatomical and biomechanical data, such as material properties and loading conditions, performing individualized FE analyses becomes feasible. Integrating FE methods with patient‐specific tailor‐made musculoskeletal models is promising in terms of contributing to the reduction and prevention of musculoskeletal disorders.

## Conclusion

Our FE model of the thoracic spine reveals asymmetric load transfer in facet joints, leading to subchondral sclerosis and hypertrophy in AIS patients. The apical facet joint of AIS helps counteract rotational stress between vertebrae and transfers most stress through the concave side. Imbalances in facet joint loading can contribute to the acceleration of scoliosis progression. The study findings contribute to our understanding of the mechanical stress loading on the facet joints of AIS patients and provide insight into the biological response of the facet joints induced by scoliotic curvature. Clarification of this phenomenon can help elucidate the mechanisms of scoliosis progression and lead to the development of new treatment strategies for AIS.

## Author Contributions

**Yasuhito Yahara:** Conceptualization; data curation; formal analysis; funding acquisition; investigation; methodology; project administration; writing – original draft; writing – review and editing. **Shoji Seki:** Investigation; methodology; project administration. **Hiroto Makino:** Investigation; methodology. **Hayato Futakawa:** Data curation; formal analysis. **Katsuhiko Kamei:** Data curation; formal analysis. **Yoshiharu Kawaguchi:** Project administration; supervision; writing – review and editing.

## Funding Information

This work was supported in part by the Grant of Japan Orthopedics and Traumatology Research Foundation Grant 448. This work was also supported in part by the Japan Society for the Promotion of Science (JSPS) KAKENHI, Grant 22H02630.

## Disclosures

The author(s) declare no conflicts of interest with respect to the research, authorship, and publication of this article.

### Peer Review

The peer review history for this article is available at https://www.webofscience.com/api/gateway/wos/peer-review/10.1002/jbm4.10812.

## Data Availability

The data supporting the results of this study are available from the corresponding author upon reasonable request.
